# Galectin-1-mediated cell adhesion, invasion and cell death in human anaplastic large cell lymphoma: Regulatory roles of cell surface glycans

**DOI:** 10.3892/ijo.2014.2319

**Published:** 2014-03-04

**Authors:** OSAMU SUZUKI, MASAFUMI ABE

**Affiliations:** Department of Diagnostic Pathology, School of Medicine, Fukushima Medical University, Fukushima 960-1295, Japan

**Keywords:** galectin-1, glycosylation, sialic acid, sialyltransferase, cell adhesion, cell death, lectin array

## Abstract

Galectin-1 is known to be one of the extracellular matrix proteins. To elucidate the biological roles of galectin-1 in cell adhesion and invasion of human anaplastic large cell lymphoma, we performed cell adhesion and invasion assays using the anaplastic large cell lymphoma cell line H-ALCL, which was previously established in our laboratory. From the cell surface lectin array, treatment with neuraminidase from *Arthrobacter ureafaciens* which cleaves all linkage types of cell surface sialic acid enhanced *Arachis hypogaea* (PNA), *Helix pomatia* (HPA) and *Phaseolus vulgaris-L* (L-PHA) lectin binding reactivity to cell surface of lymphoma cells suggesting that neuraminidase removes cell surface sialic acid. In cell adhesion and invasion assays treatment with neuraminidase markedly enhanced cell adhesion to galectin-1 and decreased cell invasive capacity through galectin-1. α2,6-linked sialic acid may be involved in masking the effect of the interaction between galectin-1 and cell surface glycans. H-ALCL cells expressed the β-galactoside-α2,6-sialyltransferase ST6Gal1. On resialylation assay by recombinant ST6Gal1 with CMP-Neu5Ac, α2,6-resialylation of L-PHA reactive oligosaccharide by ST6Gal1 resulted in inhibition of H-ALCL cell adhesion to galectin-1 compared to the desialylated H-ALCL cells. On knockdown experiments, knockdown of ST6Gal1 dramatically enhanced cell adhesion to galectin-1. N-glycosylation inhibitor swainsonine treatment resulted in enhancement of cell adhesion to galectin-1. In glycomic analysis using the lectin blocking assay treatment with PNA, *Artocarpus integrifolia* (Jacalin), *Glycine max* (SBA), *Helix pomatia* (HPA), *Vicia villosa* (VVA), *Ulex europaeus* (UEA-1), *Triticum vulgaris* (WGA), *Canavalia ensiformis* (ConA), *Phaseolus vulgaris-L* (L-PHA), *Phaseolus vulgaris-E4* (E-PHA), *Datura stramonium* (DSA) lectins resulted in modulation of lymphoma cell to galectin-1 suggesting that several types of glycans may regulate cell adhesion to galectin-1 by steric hindrance. The adhesive capacity of H-ALCL cells is regulated by phosphatidylinositol 3 phosphate kinase (PI3K) and actin cytoskeleton, and the invasive capacity of H-ALCL cells is regulated by PI3K, mitogen-activated protein kinase (MAPK), Rho and actin cytoskeleton. Furthermore, galectin-1-induced cell death in H-ALCL cells was accompanied by inhibition of CD45 protein tyrosine phosphatase (PTP) activity. In conclusion, cell adhesion and invasion to galectin-1 appeared to be regulated by cell surface sialylation and N-glycosylation, and galectin-1 regulates cell death through inhibition of CD45 PTP activity of H-ALCL.

## Introduction

Galectin-1 is expressed in anaplastic large cell lymphoma (ALCL) as well as Hodgkin lymphoma (HL) (1). However, the biological significance of galectin-1 still remains unclear. Galectin-1 is known to induce cell death in human lymphoma and T cells (1–3). Galectins act as many biological functional molecules (4). Galectin-1 exists in serum and deposits to extracellular matrix (5). In addition, galectin-1 regulates cell adhesion of ovarian cancer cells (6). Cell surface sialic acid appeared to modulate cell adhesive capacity of lymphoma cells (7). In this study, we analyzed cell adhesive and invasive capacity to galectin-1 using human ALCL cell line to clarify the biological roles of galectin-1 in ALCL. We applied the cell surface lectin array in analysis of cell surface glycan modifications (8).

## Materials and methods

### Cell line

Human anaplastic large cell lymphoma cell line, H-ALCL was established in our laboratory. The H-ALCL cells were grown in the culture medium of RPMI-1640 containing 15% fetal calf serum in 5% CO_2_ at 37°C. The H-ALCL cell line expresses the galectin-1 receptors, CD45RA [(leukocyte common antigen (LCA)] and CD45RO (UCHL-1) on the flow cytometric analysis (data not shown).

### Cell surface lectin array analysis

We applied the cell surface lectin array analysis to detecet the cell surface glycosylations according to Landemarre *et al* with several modifications (8). The H-ALCL cells were treated with or without neuraminidase from *Arthrobacter ureafaciens* (no. 10269611001, Roche, Germany) at 0.2 U/ml, at 37°C for 30 min, then the cells were cytospun and cytospin cell preparations were stained by PNA lectin as described previously (9). *Arachis hypogaea* (PNA), *Artocarpus integrifolia* (Jacalin), *Glycine max* (SBA), *Helix pomatia* (HPA), *Vicia villos*a (VVA), *Ulex europaeus* (UEA-1), *Triticum vulgaris* (WGA), *Canavalia ensiformis* (ConA), *Phaseolus vulgaris-L* (L-PHA), *Phaseolus vulgaris-E4* (E-PHA), *Datura stramonium* (DSA) lectins were from EY Laboratory. The 96-well plate was coated by each lectin and air-dried. The neuraminidase treated or non-treated H-ALCL lymphoma cells (1×10^6^/2 ml) were applied to each well (100 *μ*l/well) and incubated at 37°C for 60 min. After aspiration of the medium, PBS was added to each well and then aspirated to remove non-adhered cells. Then 100 *μ*l of 3.7% formaldehyde was added to each well to fix the adhesive cells at RT for 40 min. After aspiration of formaldehyde, 100 *μ*l of 0.1% crystal violet was added to each well and the plates were incubated at RT for 40 min. After washing twice, 100 *μ*l of 10% acetic acid was added to each well and the absorbance at 570–655 or 570 nm was determined using an ELISA plate reader (7). To analyze the effect of O-glycosylation cells were treated with the O-glycosylation inhibitor benzyl 2-acetamido-2-deoxy-3-O-β-D-galactopyranosyl -α-D-galactopyranoside (benzyl-α-GalNAc B5019, Sigma) (BZ) at a concentration of 2 mM in culture medium for 72 h at 37°C. To analyze the effect of N-glycosylation cells were treated with the N-glycosylation inhibitor SW at a concentration of 1 *μ*g/ml in culture medium for 96 h at 37°C. BZ treated or non-treated H-ALCL lymphoma cells (1×10^6^/2 ml) were applied to each well (100 *μ*l/well), or SW treated or non-treated H-ALCL lymphoma cells (2×10^6^/2 ml) were applied to each well (100 *μ*l/well) and incubated as described above.

### Cell adhesion assay

Tissue culture plates with 96-wells were coated with bovine splenic galaptin (a synomym of galectin-1, Sigma, G8777) (10 *μ*g/well) or human recombinant galectin-1 (ATGP0385, ATGen Co. Ltd.) (10 *μ*g/well) and dried at room temperature overnight. Each well was washed with 100 *μ*l PBS and was filled with RPMI-1640 containing 15% bovine serum albumin (BSA) 15% FCS and the plates were incubated at 37°C for 60 min. After aspiration of the medium, H-ALCL cells with or without neuraminidase (from *Arthrobacter ureafaciens* (AU): final concentration 0.2 U/ml, at 37°C for 30 min, α2,3-neuraminidase (BioLabs, P0728S, 50,000 U/ml): final concentration 0.2 U/ml, at 37°C for 30 min, neuraminidase from Newcastle disease virus (NDV): 0.2 U/ml, Prozyme, at 37°C for 30 min) treatment were added to each well and incubated at 37°C for 1 h. After aspiration of the medium, PBS was added to each well and then aspirated to remove non-adhered cells. Then 100 *μ*l of 3.7% formaldehyde was added to each well to fix the adhesive cells at RT for 40 min. After aspiration of formaldehyde, 100 *μ*l of 0.1% crystal violet was added to each well and the plates were incubated at RT for 40 min. After washing with PBS, 100 *μ*l of 10% acetic acid was added to each well and the absorbance at 570–655 nm or 570 nm was determined using an ELISA plate reader (7). To analyze the effect of O-glycosylation and N-glycosylation cells are treated with O-glycosylation inhibitor BZ and N-glycosylation inhibitor SW as described above. To analyze the steric hindrance of N-glycosylation in cell adhesion to galectin-1, we performed lectin blocking assay in galectin-1 adhesion assay. To analyze involvement of anaplastic large cell lymphoma kinase (ALK), CD30, CD45, CD45RO, epithelial membrane antigen (EMA) in cell adhesion to galectin-1, we carried out the inhibition assay using anti-ALK (ALK1, Dako, M7195), CD30 (BerH2, Dako, M0751), CD45 [leukocyte common antigen (LCA), Nichirei, H0108, Japan], CD45RO (UCHL-1, Dako, M0742), EMA (E29, Dako, M0613) antibody on galectin-1 adhesion assay. For isotype control experiment, the mouse IgG isotype control antibody (BD Pharmingen, no. 554721) was used. These antibodies were used as 5 *μ*l in 1 ml cell suspension. ALK, CD30, CD45, CD45RO and EMA are expressed on the cell surface of H-ALCL cells determined by flow cytometric analysis (data not shown). Anti-CD30, CD45, CD45RO and EMA antibodies enhanced cell adhesion to galectin-1 suggesting that the protein portion of these molecules may be involved in the interaction between galectin-1 (in our preliminary data, not shown).

On the resialylation assay, the H-ALCL cells were desialylated by neuraminidase treatment and then, the cells were resialylated by recombinant ST6Gal1 (R&D Systems, 5924-GT) at a condition ST6Gal1 5 *μ*g/100 *μ*l with CNP-Neu5Ac (Sigma, C8271) 1 mM, at 37°C for 120 min.

### Knockdown of ST6Gal1

In order to analyze the regulatory mechanism of cell surface sialylation by ST6Gal1, siRNA transfection was performed as described previously with several modifications (25). For transfection, INTERFERin (Polyplus transfection, USA) was used according to the manufacturer’s instructions. For knockdown experiments, siRNA (cat. no. 12842 called Type 42, sense: AGACAGUUUGUACA AUGAAtt, antisense: UUCAUUGUACAAACUGUCUtt, or cat. no. s12843 called Type 43, sense: ACCACUCAGAUAU CCCAAAtt, antisense: UUUGGGAUAUCUGAGUGGUat, Ambion Japan) was used. For control experiments, Ambion Silencer™ select negative control no. 1 siRNA (cat. no. 4390843) was applied. After 24-h incubation, the immunohistochemical staining was performed by anti-ST6Gal1 antibody (dilution ×100, R&D Systems, AF5924) and knockdown effect was validated by inhibition of ST6Gal1 protein expression in the cytoplasm of H-ALCL cells (9).

### Invasion assay

The invasion assay (haptotaxis) was performed based on the methods of Albini *et al* (10) with several modifications. The 24-well culture plate was filled with 600 *μ*l the culture medium RPMI-1640 containing 15% BSA 15% FCS. The lower surfaces of the membranes of transwell chamber, chemotaxicell (Krabo, Japan) with an 8-*μ*m pore membrane were coated with 10 *μ*l galectin-1 (1.0 mg/ml, ATGen, no. ATGP0385) and dried at RT. Then coated chemotaxicells were inserted into each well. H-ALCL cells, 100 *μ*l of 2.4×10^6^/ml, were inserted into each chemotaxicell and incubated at 37°C for 24 h. After incubation, the invaded cells at lower level of each well were counted by trypan-blue exclusion methods. The cell count was performed using duplicate wells with two experiments (n=4), or triplicate wells with at least two independent experiments. On preliminary assay the amount of invaded cells in non-coating chemotaxicell was more than that in galectin-1 coating chemotaxicell (data not shown) suggesting that H-ALCL cells can invade to lower chamber through cell ontact to galectin-1 matrix component. To analyze the effect of cell surface sialylation, H-ALCL cells were treated with neuraminidase from *Arthrobacter ureafaciens* (AU) (final concentration 0.2 U/ml) at 37°C for 30 min. For analysis of phosphatidylinositol 3 kinase (PI3K) inhibitor, wortmannin (681675, Calbiochem) and mitogen-activated protein kinase (MAPK) inhibitor, PD98059 (513000, Calbiochem) or Rho inhibitor (C3 transferase) cells were pre-incubated with wortmannin at 1.7 *μ*M or PD98059 at 25 *μ*M for 30 min, or C3 transferase at 2.0 *μ*g/ml, 2 h. Then the cell adhesion assay or invasion assay were performed. We confirmed the expression of PI3K, MAPK and Rho in the tumor cells of H-ALCL on immunohistochemical staining (data not shown). For analysis of cytochalasin B (Sigma), cells were pre-treated with cytochalasin B at 4 *μ*M for 30 min.

### Galectin-1 and ST6 Gal1 expression

H-ALCL cells were cytospun to the slide glass and the specimen was fixed with 100% alcohol. The immunohistochemical staining by anti-galectin-1 antibody (100X dilution, Santa Cruz, clone S-14) or anti-ST6Gal1 antibody (100X dilution, R&D Systems, AF5924). Then, the preparations were incubated with biotinylated anti-goat immunoglobulin. After washing in PBS three times, the preparations were incubated at room temperature for 20 min with avidin-biotin peroxidase complex kit (Dako, Tokyo, Japan). Then, they were incubated for 5 min at room temperature with diaminobenzidine (DAB)-H_2_O_2_ solution (60 *μ*g DAB in 150 ml PBS). The preparations were counterstained with hematoxylin and mounted.

### Galectin-1 detection by ELISA

The supernatant of the conditioned culture medium in H-ALCL was prepared by centrifugation at 11,000 rpm, for 20 min, at 4°C. Galectin-1 was measured by ELISA assay kit (USCN Life Science Inc. E9032 Hu) according to the manufacturer’s instructions.

### Cell death induction and CD45 activity assay by galectin-1 treatment

The H-ALCL cells were treated with or without 12 *μ*M galectin-1 for 72 h. Then the number of viable cells was counted by trypan-blue exclusion test. We analyzed the CD45 protein tyrosine phosphatase (PTP) activity by the CD45 PTP drug discovery kit, AK-812 (Biomol) according to the manufacturer’s instructions with modification. For analysis, the cell lysate was prepared by 1% NP-40 PBS buffer. The PTP inhibitor, sodium orthovanadate (V) (vanadate) (Wako, Japan) treatment was performed at 100 *μ*M for 16 h at 37°C using H-ALCL cell line for cell death analysis, and 400 *μ*M for 2 h for CD45 PTP activity analysis. The inhibition of CD45 activity by vanadate was validated on CD45 activity assay using pure CD45 protein mixed with vanadate (data not shown).

## Results

### Sialylation and cell adhesion assay

**Figure 1.**
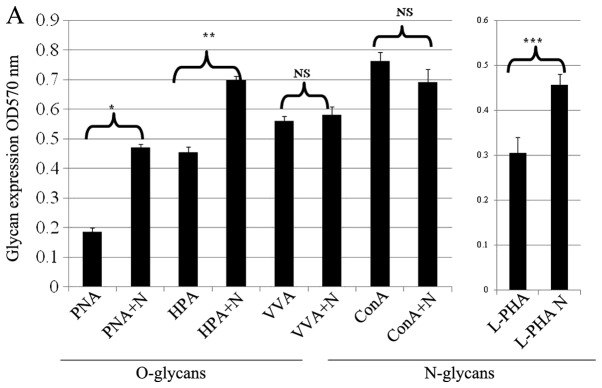

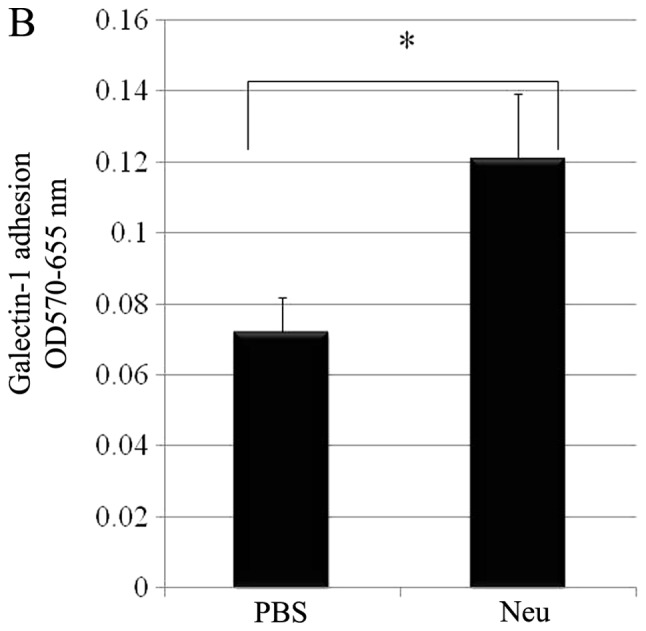

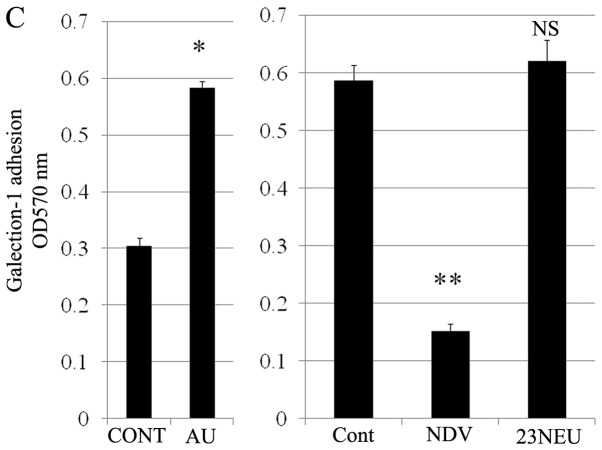

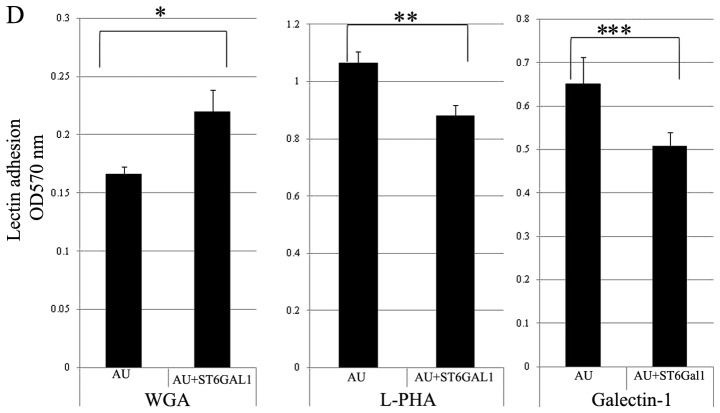

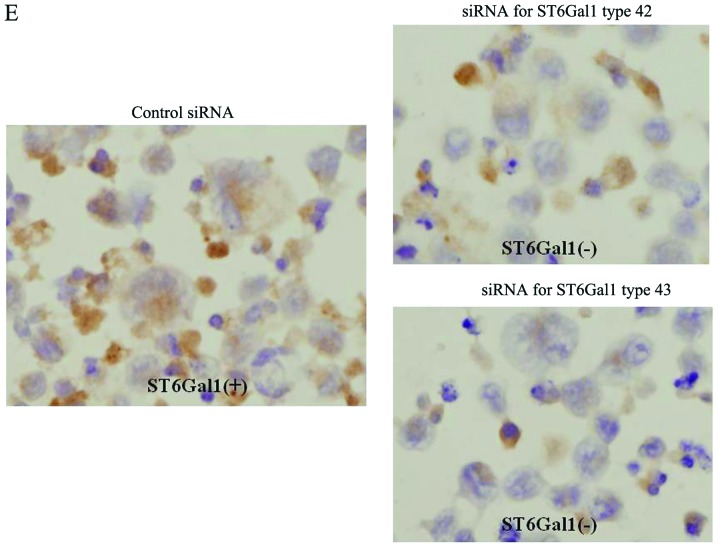

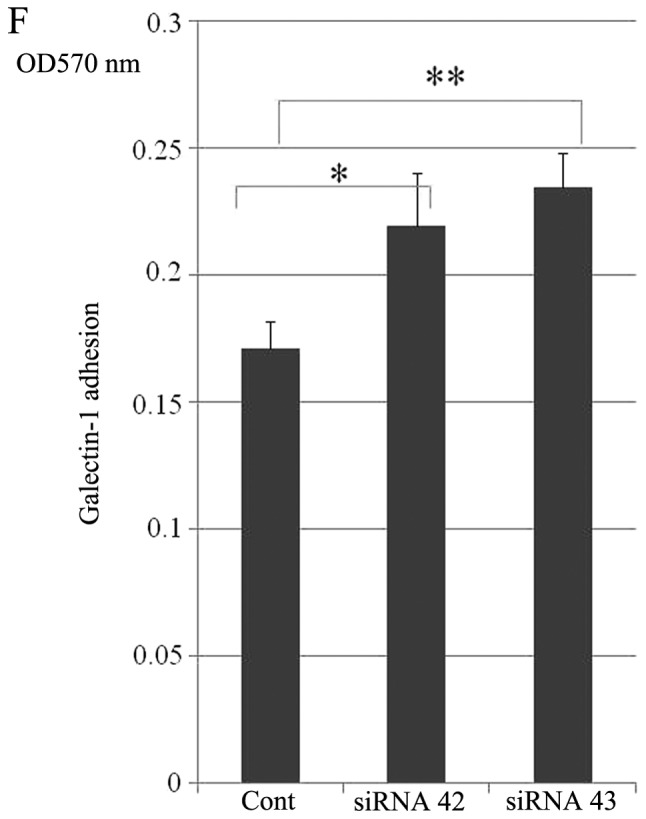
The treatment of neuraminidase which cleaves cell surface sialic acid enhanced PNA, HPA and L-PHA lectin reactivity suggesting that neuraminidase removes cell surface sialic acid from O- or N-glycans (PNA, ^*^P<0.00001; HPA, ^**^P<0.0001; L-PHA, ^***^P=0.002). N, neuraminidase pre-treatment (A). Treatment of neuraminidase markedly enhanced cell adhesion to galectin-1 (using galaptin) (^*^P<0.0009). Neu, neuraminidase pre-treatment (B). Treatment of neuraminidase from *Arthrobacter ureafaciens* markedly enhanced cell adhesion to galectin-1 (using recombinant galectin-1). Treatment of Newcastle disease virus neuraminidase dramatically inhibited cell adhesion to galectin-1 and α2,3 neuraminidase did not enhance cell adhesion to galectin-1 (C) (^*^P=0.0000069; ^**^P= 0.0001; NS, not significant). AU, neuraminidase from *Arthrobacter ureafaciens*; NDV, neuraminidase from Newcastle disease virus; 23 NEU, α2,3 specific neuraminidase. On resialylation assay, ST6Gal1 enhanced cell adhesion to WGA, and inhibited the desialyated H-ALCL cells binding capacity to L-PHA lectin and galectin-1 (^*^P=0.003; ^**^P=0.002; ^***^P=0.01) (D). On knockdown experiments, expression of ST6Gal1 on the cytoplasm of H-ALCL cells immunohistochemically (E) and knockdown of ST6Gal1 dramatically enhanced cell adhesion to galectin-1 (^*^P=0.018; ^**^P=0.0017) (F). Representative results from two or three independent experiments in triplicate are shown.

Treatment of neuraminidase which cleaves cell surface sialic acid enhanced PNA, HPA and L-PHA lectin reactivity suggesting that neuraminidase removes cell surface sialic acid from O- and N-glycans (Fig. 1A). Treatment of neuraminidase from *Arthrobacter ureafaciens* markedly enhanced cell adhesion to galectin-1 (using galaptin) (Fig. 1B). Treatment of neuraminidase from *Arthrobacter ureafaciens* markedly enhanced cell adhesion to galectin-1 (using recombinant galectin-1). Treatment of neuraminidase from Newcastle disease virus inhibited cell adhesion to galectin-1 and α2,3-neuraminidase did not enhance cell adhesion to galectin-1 (Fig. 1C). On resialylation assay, ST6Gal1 enhanced cell adhesion to WGA, and inhibited the desialyated H-ALCL cell binding capacity to L-PHA lectin and galectin-1 (Fig. 1D). On knockdown experiments, ST6Gal1 dramatically disappeared in the cytoplasm of H-ALCL cells and knockdown of ST6Gal1 enhanced cell adhesion to galectin-1 (Fig. 1E and F).

### O-glycosylation inhibitor and cell adhesion assay

**Figure 2.**
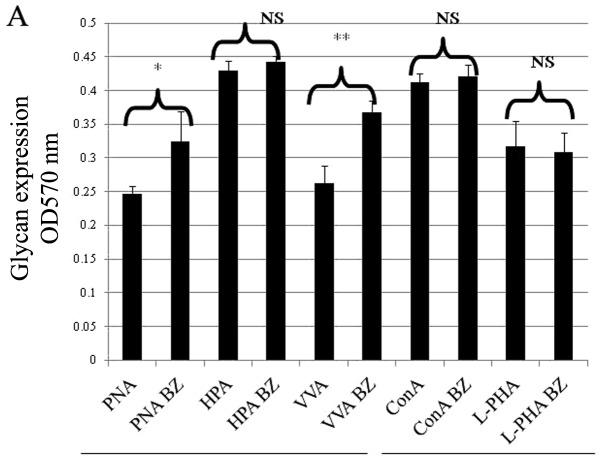

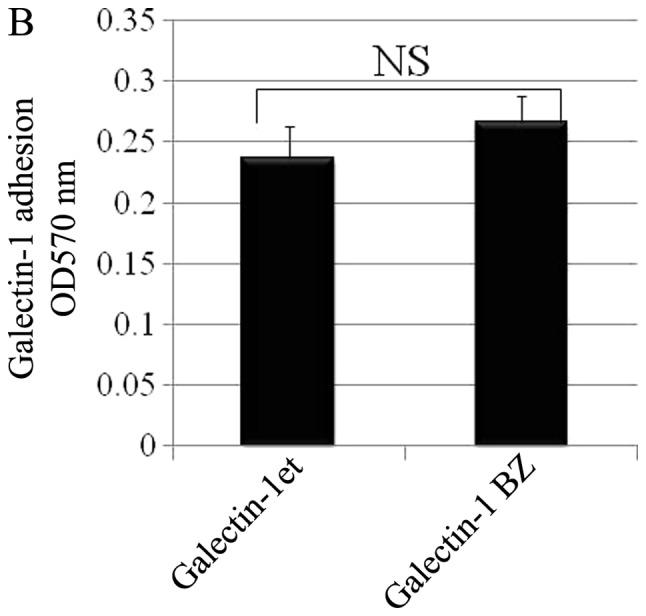
O-glycosylation inhibitor, benzyl-GalNAc (BZ) treatment resulted in the enhancement of PNA and VVA lectin reactivity suggesting the inhibition of elongation of O-glycosylation (PNA, ^*^P<0.05; VVA, ^**^P<0.005; NS, not significant) (A). ConA and L-PHA lectin binding activity which is related to N-glycans was not dramatically changed. Treatment of BZ did not show alteration of cell adhesive capacity to human recombinant galectin-1 (NS, not significant; et, ethanol control; BZ, benzyl-GalNAc) (B). Representative results from two independent experiments in triplicate are shown.

O-glycosylation inhibitor, benzyl-GalNAc (BZ) treatment resulted in the enhancement of PNA and VVA lectin reactivity suggesting the inhibition of elongation of O-glycosylation (Fig. 2A). ConA and L-PHA lectin binding activity which is related to N-glycans was not dramatically changed. Treatment of BZ did not show alteration of cell adhesive capacity to human recombinant galectin-1 (Fig. 2B).

### N-glycosylation inhibitor and adhesion assay

**Figure 3.**
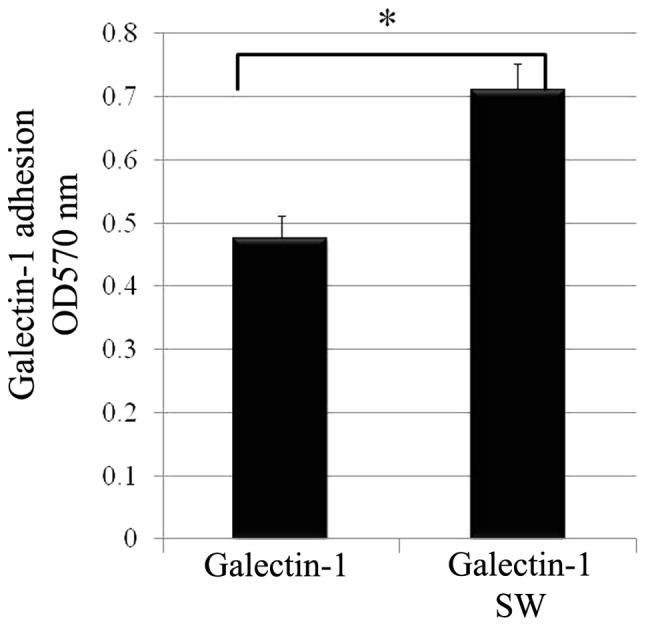
Treatment of SW markedly enhanced the cell adhesive capacity to human recombinant galectin-1 (^*^P=0.000893). Representative results from two independent experiments in triplicate are shown.

Treatment of SW markedly enhanced the cell adhesive capacity to human recombinant galectin-1 (Fig. 3).

### Glycomic analysis on galectin-1 cell adhesion assay

**Figure 4.**
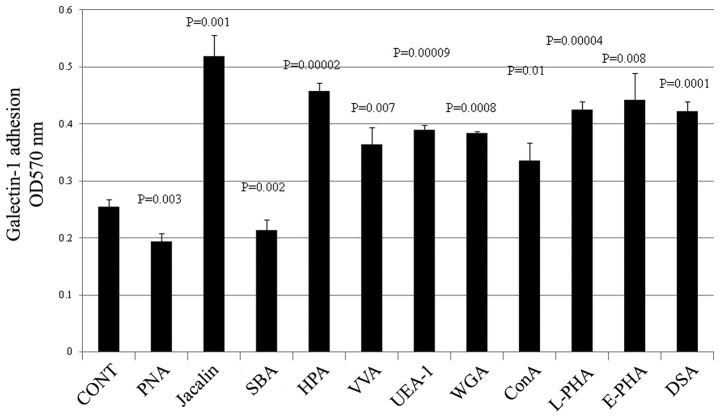
The H-ALCL cells were treated with neuraminidase from AU. The H-ALCL cells were treated with PNA, Jacalin, SBA, HPA, VVA, UEA-1, WGA, L-PHA, E-PHA and DSA lectins and these lectins modulate the adhesive properties to galectin-1 of H-ALCL cells (PNA, P=0.003; Jacalin, P=0.001; SBA, P=0.002; HPA, P=0.00002; VVA, P=0.0075; UEA-1, P=0.00009; WGA, P=0.0008; L-PHA, P=0.00004; E-PHA, P=0.008; DSA, P=0.0001; NS, not significant). Representative results from two independent experiments in triplicate are shown.

Briefly the H-ALCL cells were treated with neuraminidase from AU. Then the H-ALCL cells were treated with PNA, Jacalin, SBA, HPA, VVA, UEA-1, WGA, L-PHA, E-PHA and DSA lectins and these lectins modulate the adhesive properties to galectin-1 of H-ALCL cells (Fig. 4).

### Sialylation regulates cell invasion through galectin-1

**Figure 5.**
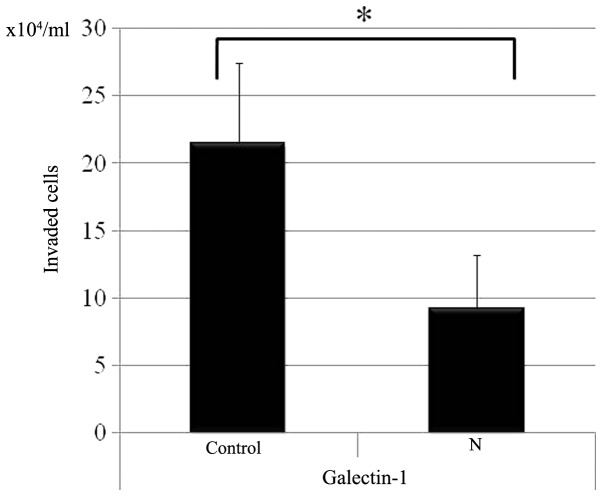
Cell invasion assay for galectin-1. The treatment of neuraminidase (N) inhibited cell invasive capacity of galectin-1 (^*^P=0.008828). Representative results from two independent experiments in triplicate are shown.

Treatment of neuraminidase markedly inhibited cell invasive capacity to galectin-1 (Fig. 5).

### Galectin-1 and ST6Gal1 expression

**Figure 6.**
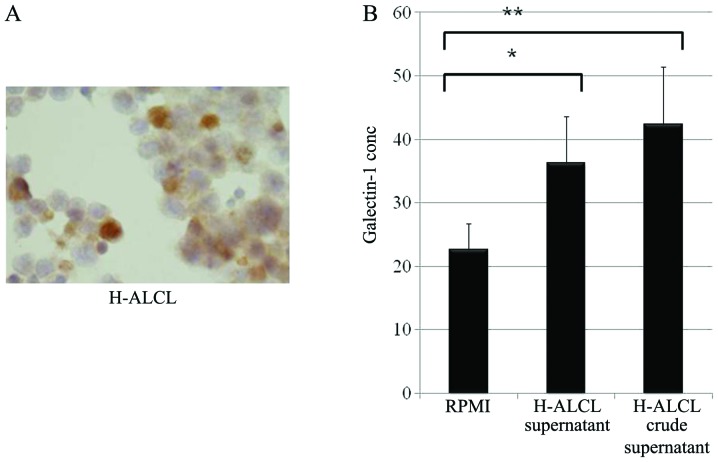

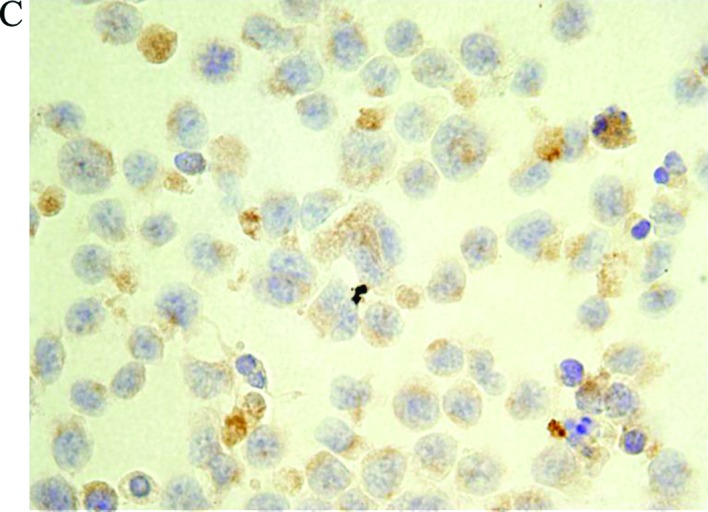
Galectin-1 and ST6Gal1 expression. (A) H-ALCL cells show galectin-1 expression in the cytoplasm on immunohistochemical analysis (magnification, ×200). (B) Galectin-1 was produced in autocrine fashion in H-ALCL cell line (^*^P=0.030705; ^**^P=0.023544). CONC, concentration. The data are a representative of two independent experiments in triplicate. (C) ST6Gal1 is expressed in the cytoplasm of H-ALCL cells (magnification, ×200).

H-ALCL cells showed galectin-1 expression in the cytoplasm (Fig. 6A). Galectin-1 was produced in autocrine fashion in H-ALCL cell line (Fig. 6B). ST6Gal1 was expressed in the cytoplasm of H-ALCL cells (Fig. 6C).

### Effect of wortmannin, PD98059, cytochalasin B and Rho inhibitor

**Figure 7.**
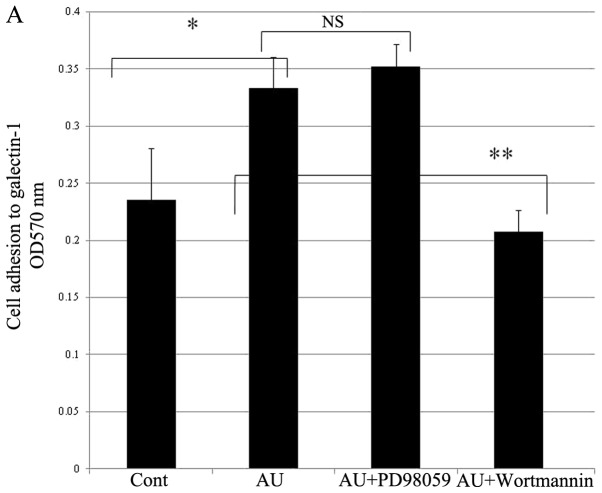

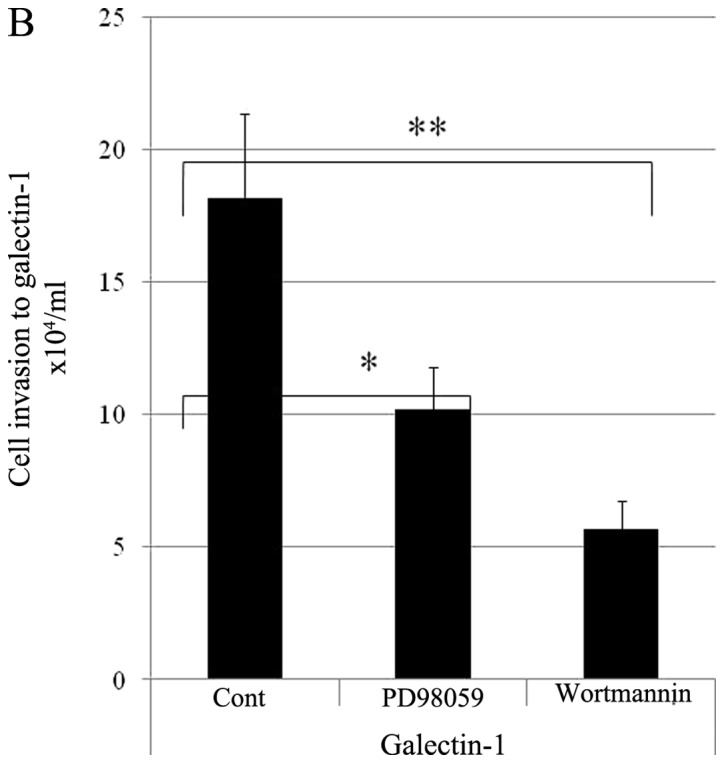

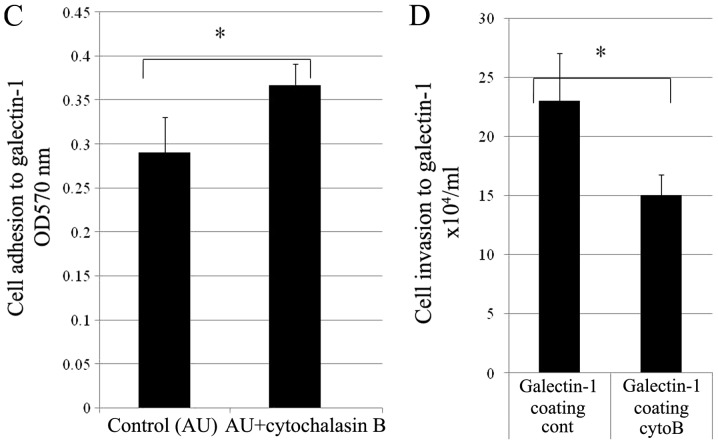

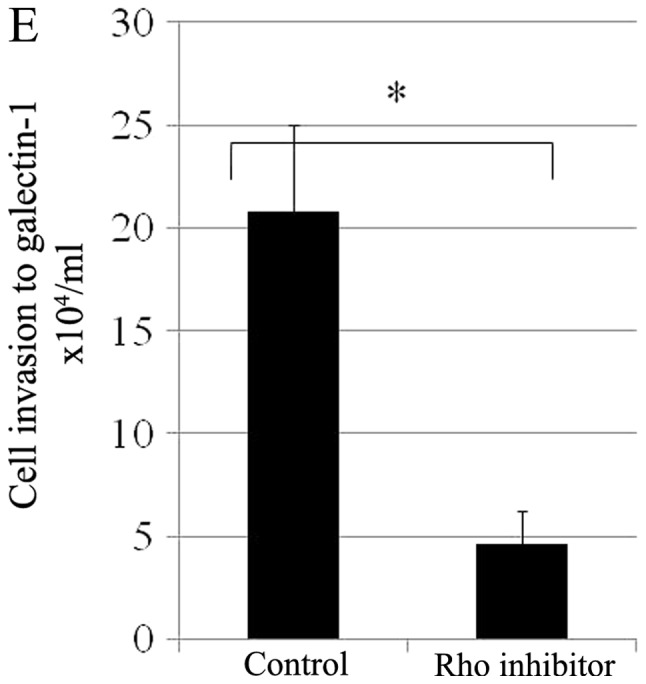
Effect of wortmannin, PD98059, cytochalasin B and Rho inhibitor. The amount of cell adhesion of H-ALCL cells was inhibited by wortmannin treatment (^*^P=0.02; ^**^P=0.002) (A). The amount of cell invasive capacity was inhibited by treatment of PD98059 and wortmannin (^*^P=0.01; s^**^P=0.0069) (B). The amount of cell adhesion of H-ALCL cells was enhanced by cytochalasin B treatment (^*^P=0.029) (C). The amount of cell invasion to galectin-1 was dramatically inhibited by treatment of cytochalasin B (^*^P=0.028) (D). The amount of cell invasion to galectin-1 was dramatically inhibited by Rho inhibitor, C3 transferase treatment (^*^P=0.006) (E). The data are representative of at least two independent experiments in triplicate.

The amount of cell adhesion of H-ALCL cells was inhibited by treatment of wortmannin (Fig. 7A). The amount of cell invasive capacity was inhibited by treatment of wortmannin and PD98059 (Fig. 7B). The amount of cell adhesion of H-ALCL cells was enhanced by treatment of cytochalasin B (Fig. 7C). The amount of cell invasion to galectin-1 was dramatically inhibited by treatment of cytochalasin B (Fig. 7D). The amount of cell invasion to galectin-1 was dramatically inhibited by treatment of Rho inhibitor, C3 transferase (Fig. 7E).

### Cell death induction by galectin-1 and CD45PTP

**Figure 8.**
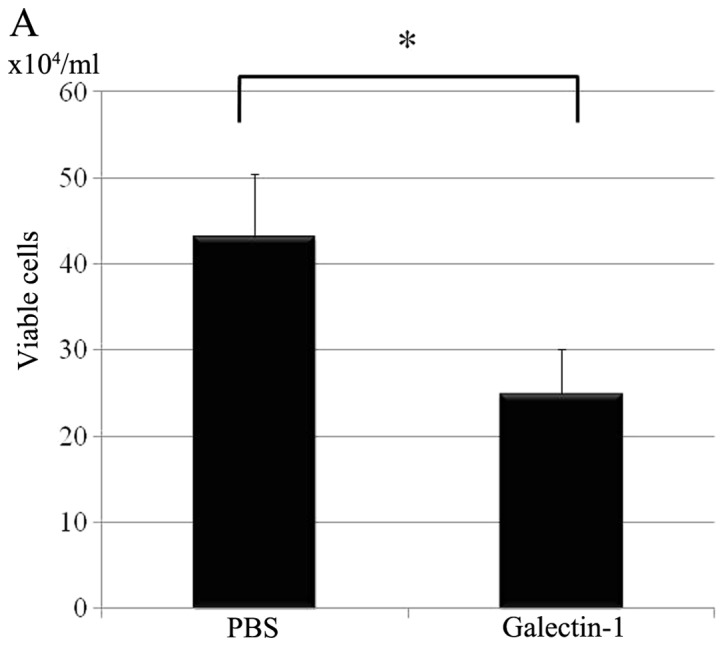

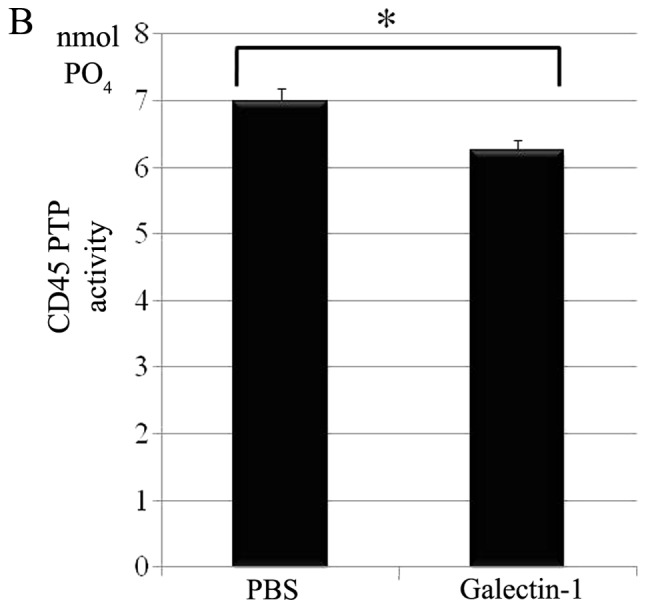

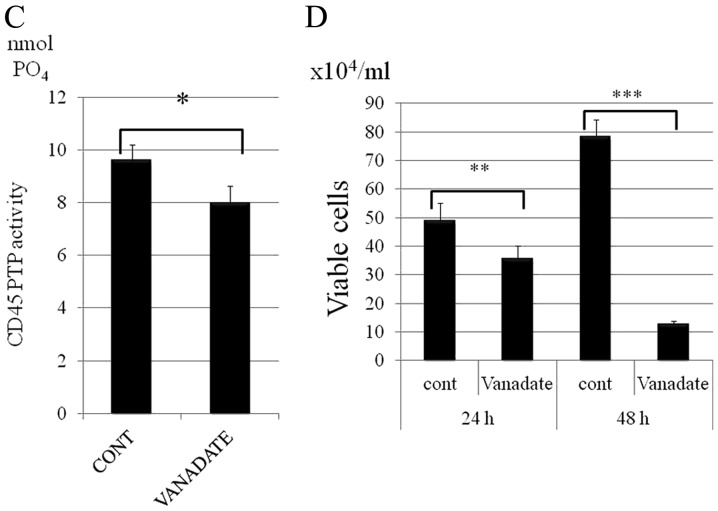
Cell death induction by galectin-1 and CD45PTP. (A) Cell death induction by galectin-1. Measurement of viable cells by trypan-blue exclusion methods revealed the number of H-ALCL cells decreased with galectin-1 treatment (^*^P=0.0014427). Representative results from two independent experiments in triplicate are shown. (B) CD45 PTP activity assay. In treatment with 20 *μ*M galectin-1 for 1 h inhibited the CD45 PTP activity (^*^P=0.003043). PO_4_, CD45 mediated release PO_4_ from unknown phosphoproteins. The data are representative of two independent experiments. (C) PTP inhibition assay. Treatment with vanadate inhibited CD45 PTP activity after 2 h at a concentration of 400 *μ*M (^*^P=0.016098; CONT, control experiment with PBS). (D) Treatment with vanadate induced cell death in H-ALCL cell line after 24 or 48 h at a concentration of 100 *μ*M (^*^P=0.021151;^**^P=0.000965). The data are representative of two independent experiments in triplicate.

Measurement of viable cells by trypan-blue exclusion method revealed the number of H-ALCL cells decreased with galectin-1 treatment after 3 days (Fig. 8A). On treatment with 20 *μ*M galectin-1 for 1 h, the CD45 PTP activity was inhibited (Fig. 8B). Vanadate can completely inhibit the recombinant CD45 PTP activity (data not shown). Treatment with vanadate inhibited CD45 PTP activity after 2 h at a concentration of 400 *μ*M (Fig. 8C) and induced cell death in H-ALCL cell line after 16 h at a concentration of 100 *μ*M (Fig. 8D).

## Discussion

Galectin-1 acts as extracellular matrix (6) as well as a modulator of cell adhesion and invasion to extracellular matrix in tumor cell lines (11–14). The present data suggested that cell surface sialylation may inhibit cell adhesion to galectin-1 in H-ALCL cells and suggested that cell surface desialylation resulted in enhancement of cell adhesion to galectin-1 and reduction in cell invasion through galectin-1. Previously we reported that α2,6-linked sialic acid is involved in masking effect on interaction between galectin-1 and cell surface glycans (7,15). In the present study α2,6-linked sialic acid is similarly involved. The data confirmed α2,6-linked sialic acid is able to modulate interactions between galectin-1 and cell surface glycans in human lymphoma. On the other hand, the neuraminidase from NDV which can cleave α2,3, 2,8, 2,9-linked sialic acids, not 2,6-linked, inhibited degree of cell adhesion to galectin-1. The α2,3 neuraminidase did not enhance cell adhesion to galectin-1. Therefore, taken together, α2,6-linked sialic acids may be essential to inhibit cell adhesion to galectin-1. Several recent reports suggested that α2,3-linked sialic acid also may be associated with tumor metastasis (16,17). In future investigations we will clarify biological functions of α2,3-linked sialic acid in malignant lymphoma. Furthermore, the H-ALCL cells expressed ST6Gal1 in the cytoplasm, and neuraminidase treatment enhanced cell adhesion to L-PHA lectin and the α2,6-linked sialylation by ST6Gal1 resulted in inhibition of cell adhesion to L-PHA lectin suggesting that ST6Gal1 resialylates the terminal galactose residue of N-glycans. Treatment with ST6Gal1 inhibits cell adhesion to galectin-1 suggesting that α2,6-linked sialylation of terminal residue of N-glycans by ST6Gal1 inhibits cell adhesion to galectin-1. The data are consistent with our previous reports using DLBCL cell line, HBL-2, as described above (7). Therefore, α2,6-sialylation of cell surface glycans may be associated with inhibitory effect of cell adhesion to galectin-1 regardless of histological types of human lymphoma cells. α2,6-sialylation by ST6Gal1 is expressed in colon cancer tissues compared to that of normal tissue and expression of ST6Gal1 is correlated to the high risk group in pediatric acute leukemia (18,19). α2,6-sialylation of tumor cells may be associated with carcinogenesis in colon cancer cells. ST6Gal1 is reported to be secreted into cell culture media (20). In our speculation a soluble form of ST6Gal1 may be a regulator to resialylate cell surface of lymphoma cells as indicated in the present results using a recombinant ST6Gal1.

In the present data neuraminidase treatment enhanced cell adhesion to PNA, HPA and L-PHA suggesting that desialylation resulted in exposure of β-galactose (ligand for PNA) or GalNAc (ligand for HPA) or lactosamine (ligand for L-PHA) residues of O- or N-glycans on the cell surface. Therefore, there is a possibility that GalNAc may influence cell adhesion to galectin-1 as well as one of the galectin-1 receptor, β-galactose residue. Cell surface sialic acid is known to be closely related to invasive capacity of lymphoma cells (21). Furthermore, the cases with the highly sialylated type glycan showed a high clinical stage in human lymphoma (9). These data suggested that the high sialylation may be associated with an advanced clinical stage of lymphoma cells which is related to invasiveness of tumor cells. From the present study galectin-1 mediated cell invasive capacity appeared to be regulated by cell surface sialylation and oversialylation may facilitate cell invasion through galectin-1. The biological role of galectin-1 such as extracellular matrix in ALCL has not yet been published. This is the first report that galectin-1 affects cell adhesion or invasion as extracellular matrix in ALCL.

The CD45 is a candidate glycoprotein of galectin-1 receptor (22). In our preliminary data from the antibody inhibition assay, we demonstrated that anti-CD30, CD45, CD45RO and EMA (one of galectin-3 receptors) antibodies enhanced cell adhesion to galectin-1 suggesting that these antibodies possess agonistic effects and the protein portions of ALK, CD30, CD45, CD45RO and EMA molecules may contribute to galectin-1 mediated cell adhesion. There is a possibility that the protein portions of glycoproteins can affect interaction between galectin-1 and cell surface glycoproteins.

On the kinase inhibitor analysis, wortmannin showed complete inhibition of cell adhesion to galectin-1, and PD98059 showed marked inhibition of invasion to galectin-1. The PI3K is known to regulate cell adhesion (23) and the MAPK is known to regulate cell migration (24). Therefore, PI3K may regulate the galectin-1-mediated cell adhesion and MAPK may regulate the galectin-1-mediated cell migration in H-ALCL cells. As cytochalasin B disrupts polymerization of actin which is a cytoskeleton, effect of cytochalasin B on cell adhesion to galectin-1 implies that cell adhesion to galectin-1 may be mediated by actin polymerization in H-ALCL cells. Rho (Ras homologue gene family member) is a protein which is associated with lymphocyte migration (25). In our present data Rho inhibitor, C3-transferase dramatically inhibits cell invasion through galectin-1. Rho may be a candidate regulator for cell migration of H-ALCL cells contacting galectin-1.

Sialylation was reported to regulate interaction between galectin-3 and ligand in tumor cells and the mechanisms are crucial in regulating adhesive and de-adhesive events in the invasive capacity of metastatic cells (26). We hypothesized that the masking effect of ligand by sialylation may be important in cell adhesion and invasion to galectins which are known to deposit in the tumor environment. Sialylation may act as switch on/off mechanism in the interaction between galectin and its oligosaccharide ligands. This hypothesis may provide a new scientific foundation in understandings the mechanisms of lymphoma cell biology, especially lymphoma cell adhesion and invasion.

Treatment of SW, a potent N-glycosylation inhibitor showed marked enhancement of cell adhesive capacity to galectin-1 suggesting that mature N-glycans which have sialylated glycans inhibit cell adhesion to galectin-1 in ALCL. Previously, we reported that SW treatment showed enhancement of lymphoma cells to galectin-1 in the human diffuse large B cell lymphoma cell line HBL-2 (7). Collectively, these data suggest that cell surface N-glycans can modulate cell adhesive capacity to galectin-1 in lymphoma cells, regardless of histological subtypes.

In eukaryotic cells the glycans mathematically show more than one million structures and due to this complexity the glycomic analysis is required to glycobiological investigation (27). In the present glycomics analysis, cell adhesion to galectin-1 was modulated by several types of lectin blocking suggesting that galectin-1-mediated cell adhesion may be regulated by several types of glycans as well as β-galactose residue. PNA, Jacalin, SBA, HPA, VVA, UEA-1, WGA, L-PHA, E-PHA and DSA lectin blocking showed modulation of cell adhesive properties to galectin-1. These findings may be supported by the hypothesis that several cell surface glycans may interfere with β-galactose by physical disturbance, such as steric hindrance. PNA and SBA lectin reactive β-galactose may be a receptor for galectin-1. On the other hand, the glycans reactive for Jacalin, UEA-1, WGA, ConA, L-PHA, E-PHA and DSA lectins may interfere the binding activity of H-ALCL cell to galectin-1 by steric hindrance. The steric hindrance by several different glycans may modulate cell adhesive properties to galectin-1. This may be a new concept of the extremely complex glycobiology.

In Hodgkin lymphoma galectin-1 is a predictive marker for evaluation of prognosis of the patients (28). The expression of galectin-1 is reported and galectin-1 may facilitate the immunosuppressive microenvironment (29,30). Previously some reports show that galectin-1 can induce apoptosis in T cells (18) and tumor galectin-1 suppresses T cell immunity through induction of apoptosis of T cells (31). Galectin-1 induced cell death is caspase-independent (32), and in our preliminary data, the cell death induced by galectin-1 in H-ALCL was caspase-independent (data not shown). In the present study galectin-1 induced cell death after 72 h. In other research, the expression of galectin-1 in ALCL and caspase-dependent apoptosis induction by galectin-1 was reported recently (1). The data implies that galectin-1 is a useful tool for therapy of ALCL. The galectin-1 receptor, CD45PTP activity was inhibited by treatment with galectin-1 in H-ALCL cells. The data are consistent with the previous reports showing the interaction between galectin-1 and CD45 in Burkitt’s lymphoma cell line (33). Our present report is the first showing interaction between galectin-1 and CD45PTP activity in ALCL cells.
